# 

**Published:** 1880-11

**Authors:** 


					﻿/Whole Numeer,
CCXXXII.
NOVEMBER, 1880.
Number 4,
Volume XX.
THE
BUFFALO
Medical and Surgical Journal.
EDITORS:
THOS. I.OTHROP, M.	A. R. DAVIDSON, M>»., -
P. W. VAN PEVMA, M. D.,	LVCIEN HOWE, M. D.,
HERMAN MYNTER, M. D.
BUFFALO :
Published by the Medical Journal Association.
Read the Notice of this Journal among the Advertisements.
Entered at the Post-Office at Buffalo, N. Y., as Second-Class Mail Matter.
				

